# Role of Interleukin-17 family cytokines in disease severity of patients with knee osteoarthritis

**DOI:** 10.1186/s42358-024-00351-5

**Published:** 2024-01-24

**Authors:** Zahra Kamiab, Hossein Khorramdelazad, Mehdi Kafi, Abdollah Jafarzadeh, Vahid Mohammadi-Shahrokhi, Zahra Bagheri-Hosseinabadi, Pooya Saeed Askari, Mitra Abbasifard

**Affiliations:** 1 Department of Community Medicine, School of Medicine, Rafsanjan University of Medical Sciences, Rafsanjan, Iran, Rafsanjan, Iran; 2 Department of Immunology, Faculty of Medicine, Rafsanjan University of Medical Sciences, Rafsanjan, Iran, Rafsanjan, Iran; 3 Student Research Committee, Rafsanjan University of Medical Sciences, Rafsanjan, Iran, Kerman, Iran; 4 Department of Immunology, School of Medicine, Kerman University of Medical Sciences, Kerman, Iran, Rafsanjan, Iran; 5 Molecular Medicine Research Center, Research Institute of Basic Medical Sciences, Rafsanjan University of Medical Sciences, Rafsanjan, Iran, Tehran, Iran; 6 Department of Clinical Biochemistry, School of Medicine, Rafsanjan University of Medical Sciences, Rafsanjan, Iran, Rafsanjan, Iran; 7 Department of Internal Medicine,, Faculty of Medicine, Molecular Medicine Research Center, Institute of Basic Medical Sciences Research, Rafsanjan University of Medical Sciences, Rafsanjan, Iran, Rafsanjan, Iran; 8 Immunology of Infectious Diseases Research Center, Research Institute of Basic Medical Sciences, Rafsanjan University of Medical Sciences, Rafsanjan, Iran, Rafsanjan, Iran; 9 Department of Internal Medicine, Ali-Ibn Abi-Talib Hospital, School of Medicine, Rafsanjan University of Medical Sciences, Rafsanjan, Iran, Rafsanjan, Iran

**Keywords:** Interleukin-17, Interleukin-25, Inflammation, Obesity, Knee osteoarthritis

## Abstract

**Background:**

Interleukin-17 (IL-17) family plays a role in the pathogenesis of knee osteoarthritis (KOA) by contributing to the inflammatory and destructive processes in the affected joint. This study aimed to measure levels of IL-17 A and IL-25 (IL-17E) in serum of KOA patients and determine their roles in the disease severity of patients.

**Methods:**

In this, 34 patients with KOA and 30 age and sex-matched healthy subjects (HS) were enrolled. Patients were categorized based on their Western Ontario and McMaster Universities Osteoarthritis Index (WOMAC), Visual Analog Scale (VAS), and Body Mass Index (BMI) scores. The enzyme-linked immunosorbent assay (ELISA) technique was employed to measure serum levels of IL-17 A and IL-25.

**Results:**

Level of IL-25 was significantly higher (*P* < 0.0001) in the KOA subjects than HS. IL-17 A level was significantly higher in KOA cases with WOMAC < 40 (*P* < 0.0001) in comparison to HS. IL-25 level was significantly higher in the KOA cases with WOMAC < 40 (*P* < 0.0001) and with WOMAC ≥ 40 (*P* < 0.0001) compared to HS. IL-17 A concentration was significantly higher in the KOA cases with VAS < 5 (*P* < 0.0001) compared to HS. IL-25 level was significantly higher in the KOA cases with VAS < 5 (*P* < 0.0001) and with VAS ≥ 5 (*P* < 0.0001) in comparison to HS. KOA patients with BMI ≥ 30 had significantly higher IL-17 A and IL-25 concentration in comparison to HS.

**Conclusions:**

The serum level of IL-25 in KOA patients is increased probably due to negative controlling feedback on inflammatory responses, which can be associated with obesity and disease activity.

## Introduction

Osteoarthritis (OA) is a condition marked by fibrosis, the formation of ectopic bone, and the destruction of cartilage [1]. Emerging evidence suggests that inflammatory responses play a role in the development of cartilage damage and the pathogenesis of OA. Specifically, the activation of chondrocytes and inflammatory cells in the articular cartilage and synovium can lead to cartilage degradation and bone remodeling. These activated cells release various mediators and enzymes that break down proteoglycans and collagen, resulting in the destruction of articular cartilage [2].

Previous studies demonstrated that the synovium fluid concentrations of pro-inflammatory cytokines, including interleukin (IL)-1, IL-6, and tumor necrosis factor- α (TNF-α), are increased in OA patients [3, 4]. Recently, a meta-analysis study showed that OA patients had significant higher serum IL-17 A levels compared to controls [5]. IL-17 A, IL-17B, IL-17 C, IL-17D, IL-17E, and IL-17 F are members of the IL-17 family [6]. IL-17 A and IL-17 F are pleiotropic and inflammatory cytokines, mainly produced by mast cells and T helper 17 (Th17) cells. IL-17 family cytokines have been suggested to be involved in the pathogenesis of several autoimmune disorders, such as rheumatoid arthritis (RA), multiple sclerosis (MS), psoriasis (PsO), and inflammatory bowel disease (IBD) and OA [7–12].

IL-25 (IL-17E) is considered as a novel member of the IL-17 family that is involved in inflammatory responses; however, its particular role remains unclear [13, 14]. It has been demonstrated that mice with IL-25 deficiency were more susceptible to experimental autoimmune encephalomyelitis (EAE) [15]. Findings have shown that following the production and secretion of the inflammatory mediators and cytokines, inflammation with varying degrees could occur in the synovium. Following this pathologic state, due to modulating the inflammatory responses and tissue destruction, anti-inflammatory cytokines such as transforming growth factor-β (TGF-β), IL-10, IL-35, and IL-25 are released from immune cells in a protective manner [16, 17]. An investigation showed that IL-25, through suppressing Th17 cell responses, plays an influential inflammation downregulating function in RA development and pathogenesis [14].

Prior research on serum levels of IL-17 A and IL-17E in KOA has not yielded an strong insight about regulation of these cytokines. To address this knowledge gap, our study was conducted with the aim of providing further insights into the roles and concentrations of these cytokines in KOA. Specifically, we focused on assessing the potential associations between IL-17 A and IL-17E and widely recognized indices of pain and disease severity, namely the Western Ontario and McMaster Universities Osteoarthritis Index (WOMAC) and the Visual Analog Scale (VAS). This investigation was conducted to elucidate the relevance of IL-17 A and IL-17E to the pathological profile of KOA patients. Moreover, recognizing the substantial impact of genetic diversity, including genetic polymorphisms, on IL-17 A and IL-17E responses, we undertook this study, marking the first of its kind within our population. By doing so, we aimed to gain a more comprehensive understanding of the roles played by these cytokines in the context of KOA specifically within our population. This approach not only addresses the novelty of our research but also underscores its necessity in providing population-specific insights into the pathogenesis and potential therapeutic targets associated with KOA.

## Methods

### Subjects

In this cross-sectional study, 34 KOA patients (mean aged 47.9 ± 5.54 years) and 30 age and sex-matched healthy subjects (HS) with a mean age of 46.5 ± 10.43 years were enrolled. Diagnosis of KOA was based on the American College of Rheumatology criteria (ACR) [18], and only subjects aging ≥ 42 years were included. Healthy subjects without any pain enrolled as controls, and subjects with a history of rheumatic disease, allergy, previous knee surgery, chronic painful condition, psychiatric disorder, inflammatory diseases, musculoskeletal disorders, kidney failure, and malignancy were excluded from the study. Additionally, we excluded individuals under treatment with anti-inflammatory or corticosteroid drugs. The applicants answered the WOMAC questionnaire to measure pain and function during daily activities [19]. Pain intensity was evaluated by a 10 cm VAS ruler graded from zero (no pain) to ten (average pain intensity during the past 48 h, during daily activities) [20]. The BMI was calculated as weight in kilograms divided by height in meters squared [21], and subjects were categorized as BMI ≤ 25 (subjects with healthy weight), 25 < BMI < 30 (considered as overweight), and BMI ≥ 30 (considered as obese). The Ethics Committee of Rafsanjan University of Medical Sciences, Rafsanjan, Iran approved this investigation (IR.RUMS.REC.1399.027). All participants were verbally informed about the research procedures, provided written documentation, and signed informed consent forms. The study protocols adhered to the ethical standards outlined in the 1975 Helsinki Declaration.

### Cytokine assay

Five mL of peripheral blood was collected from the studied patients and HS, and serums were isolated by centrifugation and stored at -80 ºC for further experiments. For measuring the cytokines serum levels, commercial IL-17 A (R&D system, USA) and IL-25 (BOSTER, USA) ELISA kits were used. The procedure was performed based on manufacturer instructions. The assay range and sensitivity of IL-17 A ELSA kit were 0.2 pg/mL-15 pg/mL and 0.051 pg/mL, respectively, and for IL-25 ELISA kit were 62.5 pg/mL-4000 pg/mL and < 10 pg/mL, respectively. The results were considered valid and included in the analysis only when the coefficients of variation (CV) for both inter-assay (between different assay runs) and intra-assay (within the same assay run) measurements were below 15% and 5%, respectively. Experiments for each sample were conducted in triplicate, meaning three technical replications.

### Statistical analysis

GraphPad Prism software version 8.0 (GraphPad Software Inc., USA) was used for data analysis and graphing. The normality of data distribution was identified by Kolmogorov–Smirnov test. The differences between studied groups were evaluated using parametric (*t*-tests) and non-parametric (Mann-Whitney) tests where applicable. Additionally, Pearson’s correlation analysis was conducted to assess a link between patients’ data. Multivariate logistic regression analysis was employed to adjust levels of cytokines for confounding factors. Data were presented as Mean ± standard deviation (SD), and a *P* value < 0.05 was considered statistically significant.

## Results

### Baseline data of the study participants

The statistical analysis showed no significant differences between KOA patients and HS regarding the age (47.9 ± 5.54, HS vs. 46.5 ± 10.43 years; *P* = 0.53) and gender (8 [26.6%] male/ 22 [73.4%] female vs. 10 [29.4%] male/ 24 [70.6%] female; *P* = 0.80). Hence, the groups were matched for age and gender. The mean of the WOMAC and VAS scores was 39.71 ± 1.31 and 5.8 ± 0.1 in KOA patients, respectively. The BMI of HS and KOA groups were 25.84 ± 3.54 and 27.42 ± 4.35 kg/m^2^, respectively, with no statistically significant difference (*P* = 0.28). The frequency of generalized and localized KOA subjects was 6 (17.6%) and 28 (82.4%), respectively (Table 1).

**Table 1:** Demographic and clinical information of KOA patients and HS group

Trait	HS (*n* = 30)	KOA (*n* = 34)	*P* value
Age (Year); Mean ± SD	47.9 ± 5.54	46.5 ± 10.43	0.53
Gender (m/f); n (%)	8 (26.6%)/ 22 (73.4%)	10 (29.4%)/ 24 (70.6%)	0.80
WOMAC; Mean ± SD	-	39.71 ± 1.31	-
VAS; Mean ± SD	-	5.8 ± 0.1	-
BMI; Mean ± SD	25.84 ± 3.54	27.42 ± 4.35	0.28
Type of OA (G/L); n (%)	-	6 (17.6%) /28 (82.4%)	-

HS: healthy subjects, WOMAC; Western Ontario and McMaster Universities Osteoarthritis Index, KOA; Knee osteoarthritis, VAS; Visual analog scale, BMI; Body mass index, G; generalized, L; localized

### Cytokine levels in KOA and HS groups

**Fig. 1:**
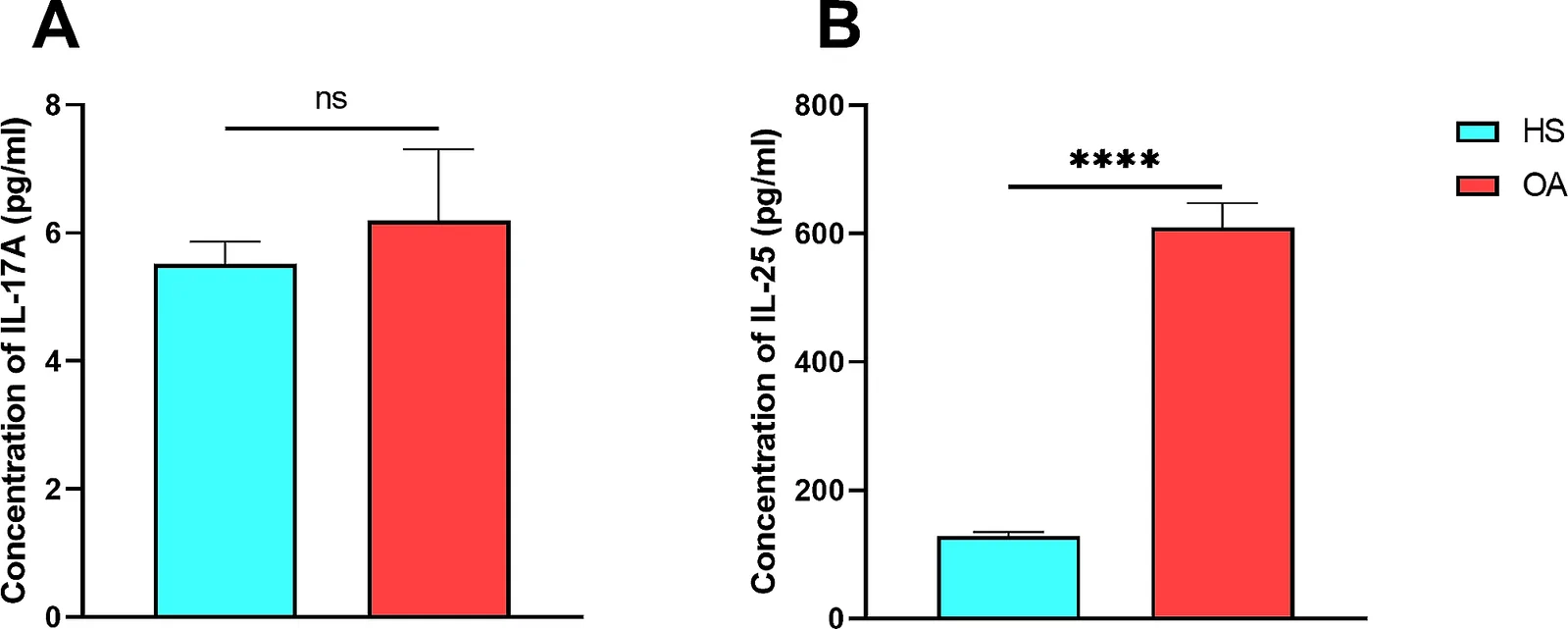
Bar graphs show the serum levels of IL-17 A **(A)** and IL-25 **(B)** in the healthy subjects and knee osteoarthritis patients. Data are shown as mean ± SD and an independent sample t-test was used to compare the groups (ns; non-significant, ****P < 0.0001)

The findings showed that there was no statistically significant difference in serum level of IL-17 A between KOA patients and HS group (5.52 ± 0.35 pg/ml vs. 6.20 ± 1.11 pg/ml; Fig. 1.A). However, serum concentration of IL-25 was significantly higher (*P* < 0.0001) in the KOA subjects compared to HS (129 ± 6.39 pg/ml vs. 610 ± 38.2 pg/ml; Fig. 1.B).

### Cytokine levels based on WOMAC score

**Fig. 2:**
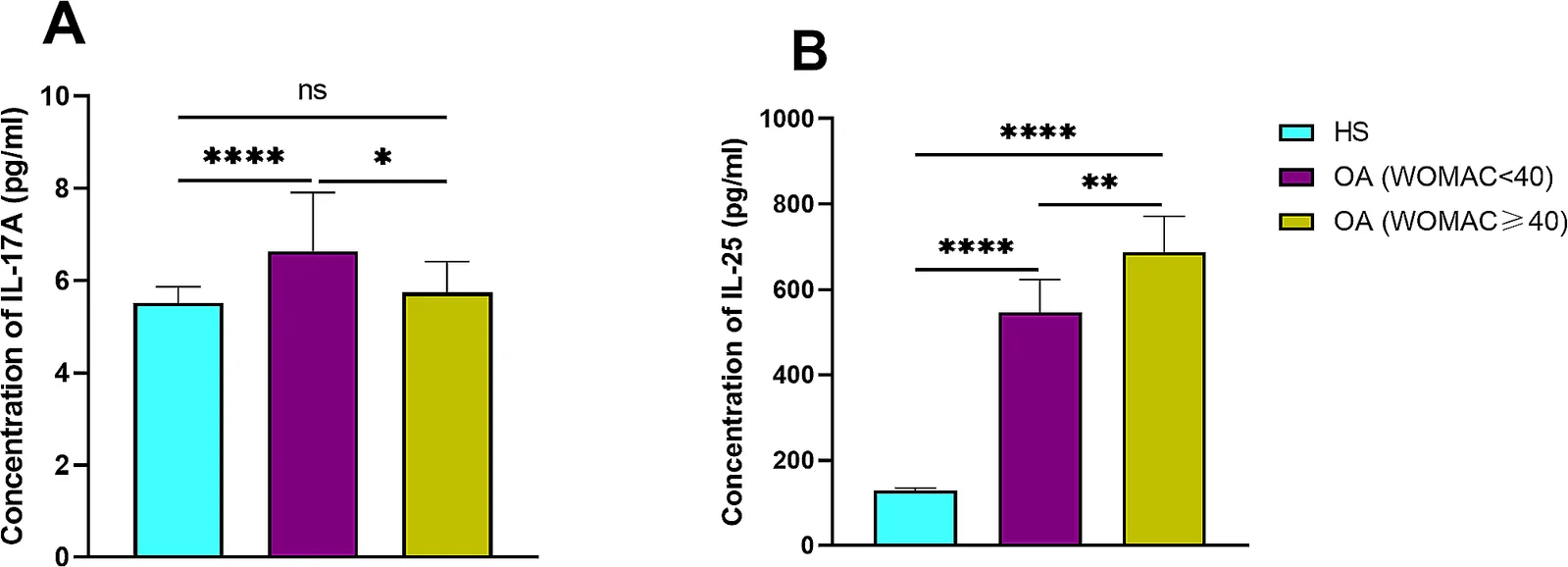
Bar graphs illustrate the serum concentration of IL-17 A **(A)** and IL-25 **(B)** in the healthy subjects and knee osteoarthritis patients with WOMAC < 40 and VOMAC ≥ 40. Data are shown as mean ± SD and an independent sample t-test was used to compare the groups (ns; non-significant, *P < 0.05, **P < 0.01, ****P < 0.0001)

IL-17 A level was significantly higher (*P* < 0.0001) in the KOA cases with MOMAC < 40 (6.64 ± 1.27 pg/ml) but not (*P* = 0.143) in KOA cases with WOMAC ≥ 40 (5.74 ± 0.68 pg/ml) in comparison to HS group (5.52 ± 0.35 pg/ml). Level of IL-17 A was significantly higher in KOA cases with WOMAC < 40 (*P* = 0.0133) in comparison to KOA cases with WOMAC ≥ 40 (Fig. 2.A). Experiments also indicated that level of IL-25 was significantly higher (*P* < 0.0001) in the KOA cases with WOMAC < 40 (547.13 ± 76.72 pg/ml) and in patients (*P* < 0.0001) with WOMAC ≥ 40 (687.69 ± 83.97 pg/ml) in comparison to HS group (129 ± 6.39 pg/ml). Additionally, level of IL-25 was significantly higher (*P* = 0.0046) in KOA cases with MOMAC ≥ 40 compared to those with WOMAC < 40 (Fig. 2.B).

### Cytokine levels based on VAS score

**Fig. 3:**
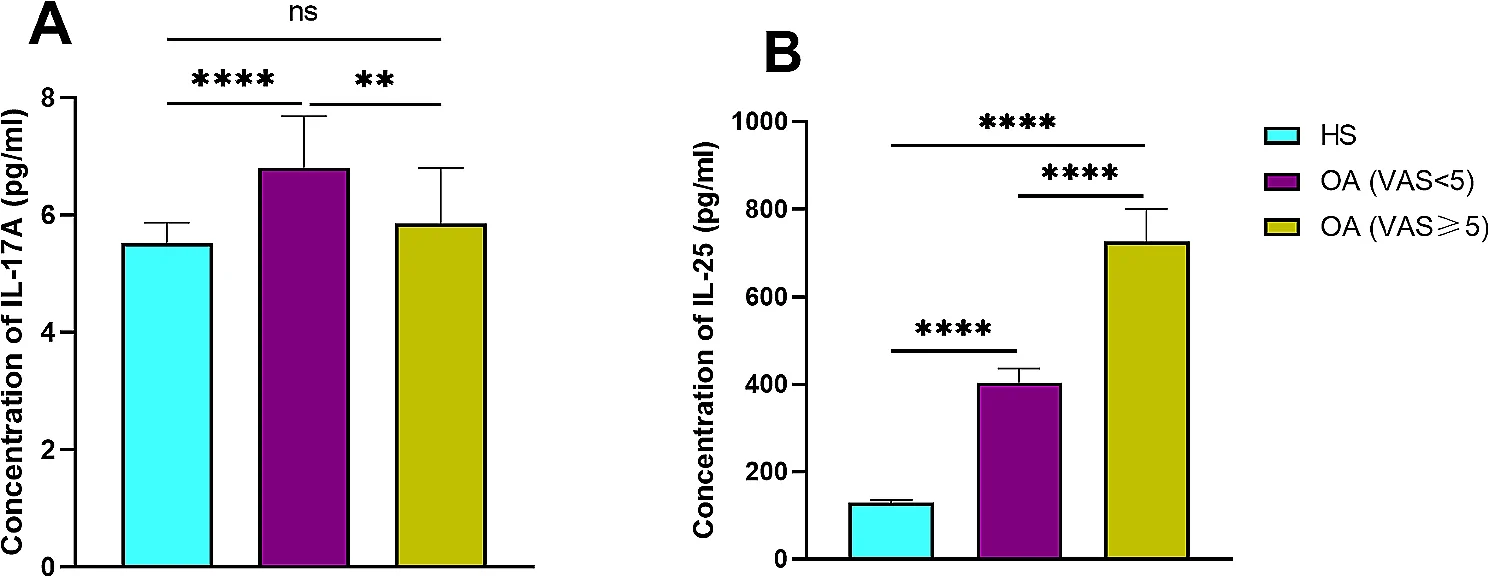
Bar graphs demonstrate the serum concentration of IL-17 A **(A)** and IL-25 **(B)** in the healthy subjects and knee osteoarthritis patients with VAS < 5 and VAS ≥ 5. Data are shown as mean ± SD and an independent sample t-test was used to compare the groups (ns; non-significant, **P < 0.01, ****P < 0.0001)

Experiments indicated that IL-17 A concentration was significantly higher (*P* < 0.0001) in the KOA cases with VAS < 5 (6.81 ± 0.87 pg/ml) but not in KOA patients (*P* = 0.477) with VAS ≥ 5 (5.86 ± 0.94 pg/ml) in comparison to HS group (5.52 ± 0.35). Level of IL-17 A was significantly higher in KOA cases with VAS < 5 (*P* = 0.0028) in comparison to KOA cases with VAS ≥ 5 (Fig. 3.A). Findings revealed that level of IL-25 was significantly higher (*P* < 0.0001) in the KOA cases with VAS < 5 (403.88 ± 31.58 pg/ml) and patients (*P* < 0.0001) with VAS ≥ 5 (752.64 ± 75.15) in comparison to HS group (129 ± 6.39 pg/ml). Also, level of IL-25 was significantly higher (*P* < 0.0001) in KOA cases with VAS ≥ 5 compared to those with VAS < 5 (Fig. 3.B).

### Cytokine levels based on BMI score

**Fig. 4:**
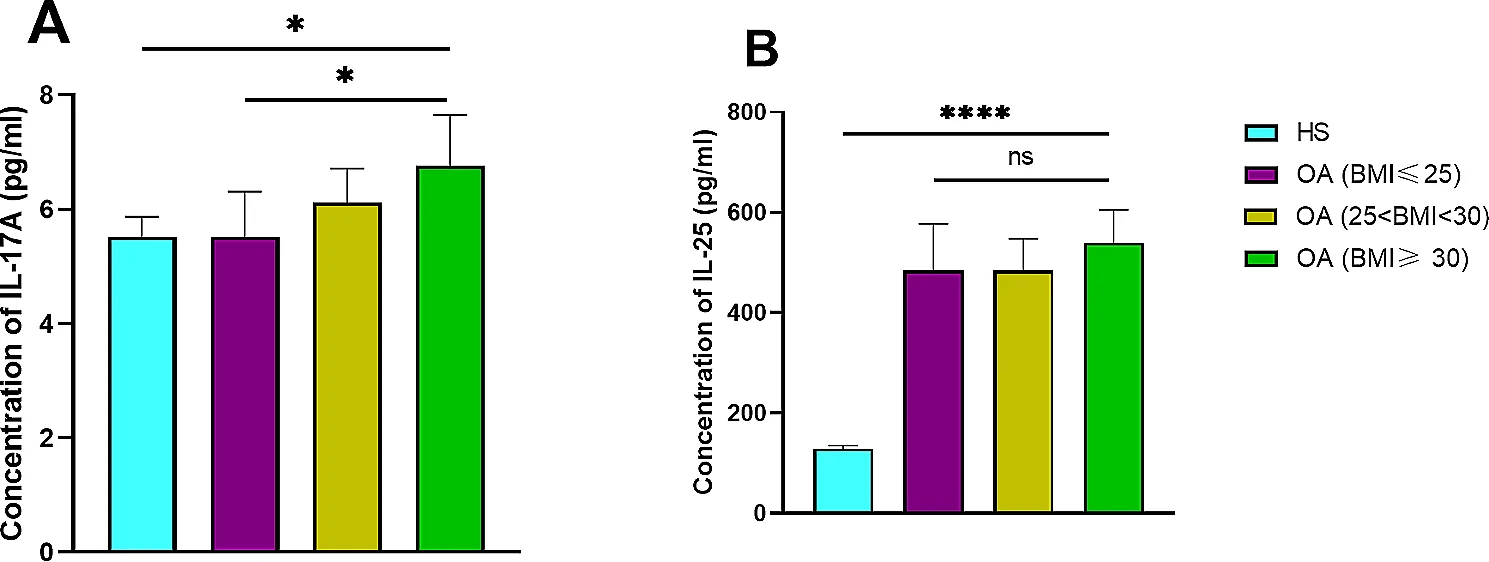
Bar graphs show the serum concentration of IL-17 A **(A)** and IL-25 **(B)** in the healthy subjects and knee osteoarthritis patients with BMI ≤ 25, 25 < BMI < 30, and BMI ≥ 30. Data are shown as mean ± SD and an independent sample t-test was used to compare the groups (ns; non-significant, *P < 0.05, ****P < 0.0001)

Results indicated that IL-17 A concentration was not significantly different between the KOA cases with BMI ≤ 25 (5.52 ± 0.79 pg/ml vs. 5.52 ± 0.36 pg/ml; *P* = 0.711) as well as 25 < BMI < 30 and HS group (6.11 ± 0.59 pg/ml vs. 5.52 ± 0.36 pg/ml; *P* = 0.355). However, KOA patients with BMI ≥ 30 had significantly higher (*P* = 0.0215) IL-17 A concentration in comparison to HS group (6.77 ± 0.81 pg/ml vs. 5.52 ± 0.36 pg/ml). Level of IL-17 A was significantly higher in KOA cases with BMI ≥ 30 (*P* = 0.011) in comparison to KOA cases with BMI ≤ 25 (Fig. 4.A).

Experiments showed that level of IL-25 was significantly higher in the serum of KOA cases with BMI ≥ 30 compared with healthy controls (541.45 ± 66.14 pg/ml vs. 129 ± 6.39 pg/ml; *P* < 0.0001). However, patients with BMI ≥ 30 did not have significant higher level of IL-25 compared to patients with 25 < BMI < 30 and patients with BMI ≤ 25. Nonetheless, level of IL-25 was significantly higher in all three BMI groups compared to HS (Fig. 4.B).

### Correlation analysis

The correlation analysis demonstrated a positive and significant correlation between the serum level of IL-17 A and IL-25 (*r* = 0.36, *P* = 0.019). The data showed no significant correlation between WOMAC and BMI scores with serum levels of IL-17 A and IL-25. There was a positive and significant correlation (*r* = 0.30, *P* = 0.041) between WOMAC and VAS in KOA subjects. In contrast, there was a positive and significant association between serum level of IL-25 (*r* = 0.33, *P* = 0.027), but not IL-17 A, and VAS in KOA patients (Table 2).

**Table 2:** Correlation analysis of cytokine levels with patients’ data

	IL-17 A	IL-25	Age	WOMAC	VAS	BMI
IL-17 A	-	*r* = 0.36, *P* = 0.019	*r* = 0.08, *P* = 0.214	*r* = 0.10, *P* = 0.180	*r* = 0.09, *P* = 0.107	*r* = 0.08, *P* = 0.251
IL-25	-	-	*r* = 0.11, *P* = 0.104	*r* = 0.08, *P* = 0.177	*r* = 0.33, *P* = 0.027	*r* = 0.07, *P* = 0.393
Age	-	-	-	*r* = 0.11, *P* = 0.094	*r* = 0.09, *P* = 0.105	*r* = 0.09, *P* = 0.209
WOMAC	-	-	-	-	*r* = 0.30, *P* = 0.041	*r* = 0.12, *P* = 0.090
VAS	-	-	-	-	-	*r* = 0.11, *P* = 0.186

IL; Interleukin, WOMAC; Western Ontario and McMaster Universities Osteoarthritis Index, VAS; Visual analog scale, BMI; Body mass index

### Regression analysis

The multivariate logistic regression analysis to control the influence of potential confounding variables (including type of OA, gender, age, WOMAC, VAS and BMI) on levels of IL-17 A and IL-25 revealed that none of the mentioned variables were affecting the comparisons. Therefore, while IL-17 A level had still non-significant difference between groups, the IL-25 level was significantly different after adjusting for confounders.

## Discussion

In this investigation, we focused on KOA and the change of the serum levels of IL-17 A and IL-25. The results demonstrated that the serum concentration of IL-17 A and IL-25 was increased in KOA patients; however, only the IL-25 concentration’s elevation was significant in KOA subjects compared with HS.

A part of our findings is parallel with the previous investigations [6, 22, 23]. Correspondingly, the findings of this study showed that KOA patients with higher WOMAC (≥ 40) and VAS (≥ 5) indexes had higher IL-25 levels than those with lower WOMAC (< 40) and VAS (< 5) index. Also, the serum level of IL-17 A in patients with a BMI of more than 30 (obese cases) was significantly higher than patients with normal BMI. These findings suggest that several factors, such as severity of the disease and metabolic and physical conditions can alter the immune responses and the levels of cytokines involved in KOA pathogenesis [24].

Several studies reported that the serum and synovium levels of IL-17 are elevated in RA and OA patients, resulting in the initiation of receptor activator of nuclear factor-κB ligand (RANK)/RANKL axis and bone and cartilage destruction [25]. Experimental studies have shown that neutralization of IL-17 by specific antibodies and other soluble inhibitors can have beneficial effects on reducing the clinical symptoms of arthritis and protecting the joints through decreasing production and release of collagen degradation factors, inflammatory cytokines, chemokines, and matrix metalloproteinases (MMPS) by osteocytes, macrophages, and synovial cells [26–29]. These factors can cause macrophages, lymphocytes, neutrophil infiltration, and inflammation in the synovium, as well as further joint damage. Accordingly, IL-17 A can cause inflammation and bone and cartilage destruction in degenerative joint diseases like KOA [30].

Subsequent to increasing disease severity and pain, inflammation and inflammatory mediators are expected to elevate [27]. In contrast, anti-inflammatory responses are anticipated to decline as several studies have previously reported that a defect in anti-inflammatory and immune-modulatory responses cause the development and progression of inflammatory disorders [31–33]. However, the results of some other studies have been contradictory [32, 34, 35]. Interestingly, results showed that IL-17 A levels was higher in patients with lower WOMAC and VAS scores, which does not align with the notion that IL-17 A might escalate the inflammation and hence the disease severity and pain. As such, since IL-25 has suppressive function on Th17/IL-17 [15], we did not observe higher IL-25 levels in patients with lower WOMAC and VAS scores, rather in those with higher scores. In addition, as IL-25 has been attributed to control and suppress the Th17/IL-17 [15], we observed that higher levels of IL-25 was correlated with higher levels of IL-17 A. However, it should be noted that a statistical comparison does not capture the full complexity of the intricate interactions that occur within biological systems.

The observed relationship between IL-25 and IL-17 levels in our study indeed raises some intriguing questions. While IL-25 is known for its ability to suppress Th17 cells and, consequently, IL-17 production [15], there might be a more complex interplay. First, the immune system is a highly intricate network where multiple factors influence each other. IL-17 regulation is not solely dependent on IL-25; other cytokines and immune responses could be involved. Second, KOA is a multifaceted condition with various immune and inflammatory components. The progression of OA involves several cytokines and immune pathways. It is plausible that the effect of IL-25 on IL-17 is overridden by the complexity of the OA pathogenesis. Third, in some cases, an increase in one regulatory factor can trigger a counter-regulatory response. It is possible that as IL-25 levels increases, the body responds by decreasing IL-17 production through alternative pathways, thus maintaining a relatively stable IL-17 level. Fourth, the timing of sample collection could be significant. A recent investigations revealed increased IL-17 A levels in serum of early KOA patients [36], whereas we did not stratify our patients based on timing of disease onset. Therefore, the relationship between IL-25 and IL-17 might be dynamic and change over time. It is possible that IL-25 levels might be initially low and as increases over the disease progresses to suppress IL-17 A.

Regarding the role of IL-25 in the articular disease pathogenesis, the latest studies revealed that IL-22-induced osteoclastogenesis could be regulated by IL-25 and expansion of IL-25 modulatory response via suppressing osteoclastogenesis may be considered as a novel and potential therapeutic strategy in the treatment of articular diseases [37]. Nevertheless, the IL-25 mechanism of action and its clinical significance in OA are not well elucidated. It was reported that levels of IL-25 were significantly higher in OA patients with more active disease than patients with lower disease activity or inactive disease [14]. Our findings also revealed that serum IL-25 level was higher in patients with higher WOMAC and VAS. As inflammatory cytokines are involved in the pathogenesis of OA, a positive correlation between serum level of IL-17 A and IL-25 was observed in the KOA patients, indicating that IL-25 may have a direct impact on Th17-mediated factors. These outcomes altogether propose that the expression and release regulatory feedback cytokines like IL-25 are induced during the OA pathogenesis, and IL-25 levels might be increased in a homeostatic manner to modulate dysregulated inflammatory responses [38].

Our experiments indicated higher IL-17 A levels in obese KOA cases (with BMI BMI) compared with overweight patients and patients with normal body weight. Furthermore, IL-17 A level in obese cases was higher than control group. These observations suggest a link between obesity and inflammation. Obesity is known to be associated with chronic, low-grade inflammation. The increased IL-17 may indicate that the pro-inflammatory environment in obese individuals is influencing the immune response in KOA. Additionally, this finding highlights the intricate interplay between obesity and KOA. Obesity is a well-established risk factor for KOA [39], but the exact mechanisms through which it contributes to the disease are not fully understood. The higher IL-17 levels in obese KOA patients could be one piece of this complex puzzle. Identifying differences in cytokine profiles based on BMI can contribute to better patient stratification. It may help in tailoring treatment approaches and interventions based on individual patient characteristics.

## Conclusion

In conclusion, the findings of this study shed valuable light on the intricate relationship between cytokines, disease severity, and obesity in KOA. The observation that KOA patients with elevated WOMAC and VAS scores exhibit higher levels of IL-25 implies a potential link between the suppression of the Th17 immune response and disease progression. Significantly, the lack of a disparity in IL-25 levels between patients with varying BMI suggests that obesity may not be a contributing factor in the modulation of IL-25 in KOA. This nuanced insight into the role of IL-25 highlights its potential as a promising therapeutic target for the treatment of KOA. One of the major concerns in our research is lower sample size, limiting the generalizability to a broader population. These results underscore the significance of further research in elucidating the precise mechanisms and therapeutic implications of cytokine modulation in KOA and pave the way for more targeted and effective interventions for this debilitating condition. These findings encourage the pursuit of research focused on unraveling the intricate web of obesity and inflammation with regard to Th17/IL-17 in KOA as well as potential regulatory function of IL-25 on IL-17 A. Further investigations in these areas have the potential to enhance our understanding of disease mechanisms and ultimately pave the way for more targeted and effective therapeutic interventions for KOA patients.

## Data Availability

The datasets used and/or analyzed during the current study available from the corresponding author on reasonable request.

## Abbreviations

KOA: Knee Osteoarthritis
IL-17: interleukin-17
HS: healthy subjects
WOMAC: Western Ontario and McMaster universities osteoarthritis index
VAS: visual analog scale
BMI: body mass index
ELISA: enzyme-linked immunosorbent assay
TNF-α: tumor necrosis factor-alpha
Th17: T helper
RA: rheumatoid arthritis
MS: multiple sclerosis
PsO: psoriasis
IBD: inflammatory bowel disease
EAE: experimental autoimmune encephalomyelitis
TGF-β: tumor growth factor-β
ACR: American College of Rheumatology criteria
